# Case of Traumatic Tetanus Successfully Treated

**Published:** 1817-08

**Authors:** Emanuel Lazzaretto

**Affiliations:** Member of the Royal College of Surgeons, London


### *Case of Traumatic Tetanus successfully treated.*

By Emanuel Lazzaretto, M.D. F.R.S. and Member of the Royal College of Surgeons, London.

On the 10th of April, John Brown, seaman, ætat. 25, complained of a pain in his right thigh, for which he desired to be put on the sick list. On inquiry, it was found that he had been intoxicated the preceding evening, and, in a scuffle with John Baptiste, a black, who was on the sick list for the cure of syphilis, received a bite in the thigh, of which, however, he took little or no notice at the time.

On examination, I found the thigh very much inflamed and swelled, with a small wound, bearing the impression of the teeth by which it had been inflicted. A cataplasm, the liquid part of which was composed of the *liq. plumb. acet. dilut.* was ordered to be applied thrice a day, and a brisk dose of the sulphate of magnesia to be administered internally.

11th. The purgative has had no effect.* He complains of stiffness and pain in the back and lower extremities, with rigidity and severe catchings of the abdominal muscles. The lower jaw is firmly clenched against the upper; and there is considerable difficulty in the deglutition of liquids. I found all the muscles round the neck hard and tense, so that he was quite unable to move his head in any direction. The pulse was 100, and the wound had a black and bad appearance. The cataplasm to be continued, and to take six grains of calomel and fifteen

---

* This circumstance, as far as an insulated fact can go, would seem to corroborate the judicious suggestions of Dr. Dickson, respecting the torpor of bowels preceding tetanus, even of the traumatic kind. The purgative administered was very powerful in this case.

grains of jalap every three hours, till the bowels were plentifully evacuated.

12th. The opening medicine has been repeated four times without any effect on the bowels. In every other respect, the patient is much worse, and all the symptoms exasperated. Pulse 120. To have diluent drinks and the saline draughts. A purgative enema to be thrown up, and at bed-time he had a full dose of laudanum and antimonial wine. The wound was treated as before, and volatile embrocations were applied to the rigid muscles of the neck, &c.

13th. Has passed a very restless night, with profuse perspirations, and some delirium. The spasms are exceedingly severe. The jaw completely locked, and the abdominal muscles drawn up into hard knots. Pulse 120. Has had only one stool, of a dark green colour, and particularly offensive smell. The wound looks black, with a sloughy fœtid discharge; the swelling and livor of the surrounding parts, however, are disappearing. To take ten grains of calomel and fifteen grains of jalap every four hours. The dose of laudanum and antimonial wine at bed-time as before. The potassa cum calce was applied to the wound; and this day the cold affusion was tried on the surface of the body.

14th. The cold affusion yesterday seemed to produce some relaxation of the tetanic spasms. He passed a better night than the preceding, and voided two liquid stools of a very green colour and disagreeable smell. The delirium is less apparent, and the pulse reduced to 110. The sore looks better, and the discharge is not so offensive. The calomel and jalap to be continued as before. The cold affusion.

15th. Has passed three copious yellow stools during the night. Spasms more moderate; countenance improving; pulse 98; wound looking better. The calomel and jalap to be continued. Cold affusion.

16th. Had five copious yellow stools, of a more natural smell; the spasms have abated considerably; copious ptyalism has taken place. He opened his mouth while asleep this morning. Has had a most profuse perspiration on the skin in the night. Discontinued the calomel and jalap, and to take ten grains of ex. col. comp. every four hours.

17th. Better in every respect. Has had three stools of nearly a natural appearance. The spasms are relaxing; the ptyalism considerable. Slept a little last night. The abdominal muscles are still rigid. The ex. colocynth continued.

18th. All the symptoms ameliorated. Complains of great anxiety about the præcordia; and has a very dejected appearance. To take infusion of cascarilla with compound spirit of ammonia, three or four times a day, with generous diet and Port wine. From this time he rapidly recovered, and was discharged to duty on the 28th of April.

His Majesty's Ship Queen Charlotte,
Portsmouth Harbour, 10th June, 1817.

---

